# A Review of the Application of Information Theory to Clinical Diagnostic Testing

**DOI:** 10.3390/e22010097

**Published:** 2020-01-14

**Authors:** William A. Benish

**Affiliations:** Department of Internal Medicine, Case Western Reserve University, Cleveland, OH 44106, USA; wab4@cwru.edu

**Keywords:** entropy, information theory, multiple diagnostic tests, mutual information, relative entropy

## Abstract

The fundamental information theory functions of entropy, relative entropy, and mutual information are directly applicable to clinical diagnostic testing. This is a consequence of the fact that an individual’s disease state and diagnostic test result are random variables. In this paper, we review the application of information theory to the quantification of diagnostic uncertainty, diagnostic information, and diagnostic test performance. An advantage of information theory functions over more established test performance measures is that they can be used when multiple disease states are under consideration as well as when the diagnostic test can yield multiple or continuous results. Since more than one diagnostic test is often required to help determine a patient’s disease state, we also discuss the application of the theory to situations in which more than one diagnostic test is used. The total diagnostic information provided by two or more tests can be partitioned into meaningful components.

## 1. Introduction

Information theory was developed during the first half of the twentieth century to quantify aspects of communication. The pioneering work of Ralph Hartley and, subsequently, Claude Shannon was primarily motivated by problems associated with electronic communication systems [1,2]. Information theory was probably first used to quantify clinical diagnostic information by Good and Card in 1971 [3]. Subsequent papers helped to clarify the ability of information theory to quantify diagnostic uncertainty, diagnostic information, and diagnostic test performance, e.g., [4,5,6,7,8,9]. Although applications of information theory can be highly technical, fundamental concepts of information theory are not difficult to understand. Moreover, they are profound in the sense that they apply to situations in which “communication” is broadly defined.

Fundamental information theory functions are defined on random variables. The ubiquity of random processes accounts for the wide range of applications of the theory. Examples of areas of application include meteorology [10], molecular biology [11], quantum mechanics [12], psychology [13], plant pathology [14], and music [15]. The random variables of interest to the present discussion are an individual’s disease state (D) and diagnostic test result (R). We require that the possible disease states be mutually exclusive and that, for each diagnostic test performed, one result is obtained. Hence, it is meaningful to talk about the probability that an individual randomly selected from a population is in a certain disease state and has a certain test result. 

The primary purpose of this review is to understand the answers that information theory gives to the following three questions:

(1) How do we quantify our uncertainty about the disease state of a given individual?
(2) After a diagnostic test is performed and a specific test result is obtained, how do we quantify the information we have received about the tested individual’s disease state?
(3) Prior to performing a diagnostic test, how do we quantify the amount of information that we expect to receive about the disease state of the tested individual?

The answers that information theory gives to these questions are calculated using pretest and posttest probabilities. Whenever the pre-test and post-test probabilities differ, the test has provided diagnostic information [16]. The functions are applicable to situations in which any number of disease states are under consideration and in which the diagnostic test can yield any number of results (or continuous results) [17]. Moreover, a given test result can alter the probabilities of multiple possible disease states.

Since information theory functions depend only upon the probabilities of states, the information content of an observation does not take into consideration the meaning or value of the states [18] (p. 8). For example, the statement that a patient died who had been given a 50-50 chance of survival contains the same amount of information, from an information theory perspective, as the statement that a tossed coin turned up heads. 

More than one diagnostic test is often required to help clarify a patient’s disease state. Hence, an additional goal of this review is to answer questions 2 and 3, above, for the case in which two or more diagnostic tests are performed. We find that it is possible to quantify both the information that we have received from each of two or more diagnostic tests as well as the information that we expect to receive by performing two or more diagnostic tests.

The foundational theorem of information theory is the statement proved by Shannon that the entropy function, discussed below, is the only function that satisfies certain criteria that we require of a measure of the uncertainty about the outcome of a random variable [2]. As an alternative to this axiomatic approach to deriving information theory functions, we employ the concept of the surprisal, with the goal of achieving a more intuitive understanding of these functions. The surprisal function is explained in the following section. It is then used in Section 3 to answer the above three questions and, in doing so, derive expressions for three fundamental information theory functions: the entropy function (Section 3.1), the relative entropy function (Section 3.2), and the mutual information function (Section 3.3). The application of information theory functions to situations in which more than one diagnostic test is performed is considered in Section 4. Section 5 provides a brief review of the history of the application of information theory to clinical diagnostic testing. Examples which offer insight into what information theory can teach us about clinical diagnostic testing are presented in Section 6. The paper concludes by briefly summarizing and clarifying important concepts. 

## 2. The Surprisal Function

The surprisal function, μ, quantifies the unlikelihood of an event [19,20]. It is a function of the probability (p) of the event. As its name suggests, it can be thought of as a measure of the amount we are surprised when an event occurs. Hence, this function assigns larger values to less likely events. Another reasonable requirement of the surprisal function is that, for independent events $a_{1}$ and $a_{2}$, the surprisal associated with the occurrence of both events should equal the sum of the surprisals associated with each event. Since $a_{1}$ and $a_{2}$ are independent, $p\left(a_{1},a_{2}\right)\;=\;p\left(a_{1}\right)p\left(a_{2}\right).$ We therefore require that $\mu \left[p\left(a_{1}\right)p\left(a_{2}\right)\right]=\mu \left[p\left(a_{1}\right)\right]+\;\mu \left[p\left(a_{2}\right)\right]$. The only non-negative function that meets these requirements is of the form:

(1) μ(p)=−log(p)

**Figure 1:**
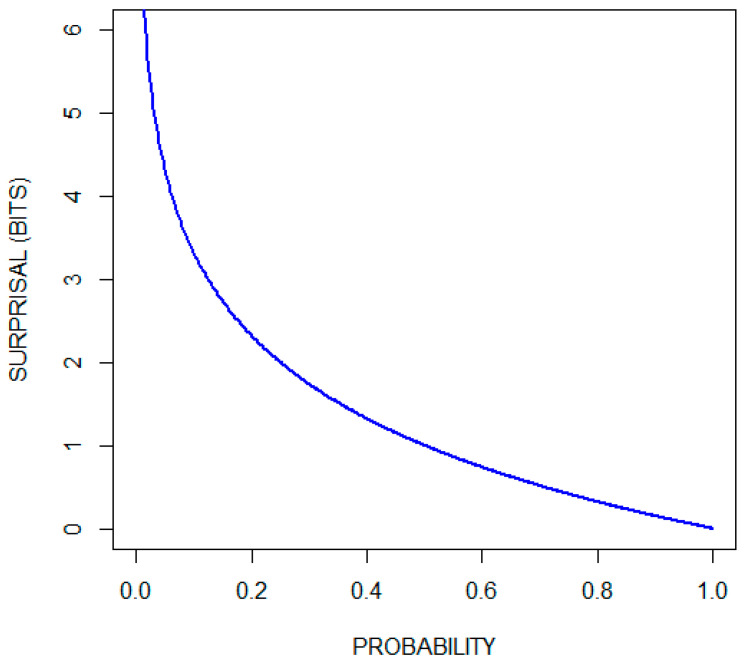
Surprisal (in bits) as a function of probability.

Ref. [21] (pp. 2–5). The choice of the base of the logarithm is arbitrary in the sense that conversion from one base to another is accomplished by multiplication by a constant. Two is often selected as the base of the logarithm, giving measurements in units of bits (binary digits). Some authors use the natural logarithm (giving measurements in units of nats) or log base 10 (giving measurements in units of hartleys) [22]. Using log base two, the surprise when a fair coin turns up heads is quantified as one bit, since $-\log_{2}\left(1/2\right)=1.$ Figure 1 plots the surprisal function (in units of bits) over the range of probabilities. Observe that the surprisal associated with the occurrence of an event that is certain to occur is zero, and that there is no number large enough to quantify the surprise associated with the occurrence of an impossible event.

## 3. Answers to the Questions Asked in the Introduction

### 3.1. Entropy Quantifies the Uncertainty about the Disease State

Suppose that the possible causes of a patient’s condition consist of four disease states, $d_{1}$, …, $d_{4},$ with respective probabilities 1/8, 1/2, 1/8, and 1/4. How uncertain are we about the disease state? The more certain we are about the disease state the less surprised we will be, on average, when the disease state becomes known. This suggests that diagnostic uncertainty be quantified as the expected value of the surprisal. For the current example, the surprisals corresponding to the four probabilities are 3 bits, 1 bit, 3 bits, and 2 bits, respectively. To calculate the expected value of the surprisal we multiply each surprisal by its probability and then sum the four terms:

$$\left(\frac{1}{8}\right)\left(3\;\mathrm{bits}\right)+\left(\frac{1}{2}\right)\left(1\;\mathrm{bit}\right)+\left(\frac{1}{8}\right)\left(3\;\mathrm{bits}\right)+\left(\frac{1}{4}\right)\left(2\;\mathrm{bits}\right)=1.75\;\mathrm{bits}.$$

This procedure yields Shannon’s entropy (H) of D, where D is the random variable associated with the four disease states. For the general case in which there are n possible disease states [2,23]:

(2) $$H\left(D\right)=-\overset{n}{\underset{i=1}{\sum }}p\left(d_{i}\right)\log_{2}p\left(d_{i}\right).$$

We saw above that the surprisal associated with a tossed coin turning up heads is 1 bit. Consequently, the uncertainty associated with the two possible outcomes of a coin toss is (1/2)(1 bit)+(1/2)(1 bit)=1 bit. The uncertainty about the outcome of equally likely events increases as the number of possible events increases; for example, the uncertainty associated with three, four, and five equally likely events is 1.59 bits, 2 bits, and 2.32 bits, respectively.

Another way to think about the meaning of entropy is in terms of the average number of yes/no questions required to learn the outcome of the random variable. This works for cases like the current example, in which, before asking each question the remaining events can be partitioned into two groups of equal probability. For the current example, we first ask if the individual is in state $d_{2}$, and then, if necessary, ask if the individual is in state $d_{4}$, and finally, if necessary, ask if the individual is in state $d_{1}$ (or state $d_{3}$). We find that, on average, we will ask 1.75 questions.

In Shannon’s axiomatic approach to the definition of the entropy function, a key requirement relates to the way in which an entropy calculation can be partitioned [18] (p. 49). As applied to the current problem, Shannon required, for example, that

$$H\left(\frac{1}{8},\frac{1}{2},\frac{1}{8},\frac{1}{4}\right)=H\left(\frac{1}{8},\frac{7}{8}\right)+\frac{7}{8}H\left(\frac{4}{7},\frac{1}{7},\frac{2}{7}\right).$$

This corresponds to first determining the entropy associated with whether the individual is in state $d_{1}$ and, if not, determining the entropy of the remaining three options. This latter entropy is weighted by 7/8, the probability that the individual is not in state $d_{1}$.

Some authors refer to entropy as self-information [23] (p. 12). In this review, we restrict the use of the term information (diagnostic information) to measures of the magnitude of changes in the probabilities of states (disease states) that result from observations (diagnostic test results).

### 3.2. Relative Entropy Quantifies the Diagnostic Information Provided by a Specific Test Result

Table 1 presents hypothetical data showing characteristics of a population of 96 individuals, each of whom is in one of four disease states and who, when tested, will yield one of three possible results. The probabilities that an individual randomly selected from this population will be in the four disease states is identical to the probabilities in the above example: 1/8, 1/2, 1/8, and 1/4, respectively. If the diagnostic test is performed and result $r_{3}$ is obtained, the respective probabilities become 1/8, 1/4, 1/2, and 1/8. Because the post-test probabilities are the same as the pretest probabilities, even though the order has changed, the uncertainty about the disease state remains 1.75 bits. Has this test provided us with diagnostic information and, if so, how much?

The test result, $r_{3}$ identifies the patient as belonging to a subset within the larger population. It provides us with diagnostic information because the probabilities of the disease states are different within this subset than they are within the larger population. We quantify diagnostic information as the expected value of the reduction in the surprisal that results from testing. To calculate the amount of information obtained from this test result, we first note that the probabilities change from

$$\left[\frac{1}{8},\frac{1}{2},\frac{1}{8},\frac{1}{4}\right]\;to\;\left[\frac{1}{8},\frac{1}{4},\frac{1}{2},\frac{1}{8}\right],\;$$

respectively; the surprisals (in units of bits) change from

[3,1,3,2] to [3,2,1,3], 

respectively; and the reductions in the surprisals (in units of bits) are

[0,−1, 2, −1],

respectively. To calculate the expected value of the reduction in the surprisal, we use the updated probabilities obtained by testing:

$$\left(\frac{1}{8}\right)\left(0\;\mathrm{bits}\right)+\left(\frac{1}{4}\right)\left(-1\;\mathrm{bit}\right)+\left(\frac{1}{2}\right)\left(2\;\mathrm{bits}\right)+\left(\frac{1}{8}\right)\left(-1\;\mathrm{bit}\right)=\frac{5}{8}\mathrm{bits}.$$

Hence, test result $r_{3}$ provides 5/8 bits of information about the disease state.

For the general case with pretest probabilities: $p\left(d_{1}\right),\;p\left(d_{2}\right),\ldots ,p\left(d_{n}\right)$ and posttest probabilities after receiving result $r_{j}$: $p(d_{1}|r_{j}),\;p(d_{2}|r_{j}),\;\ldots ,p(d_{n}|r_{j})$, the reduction in the surprisal for the *i*-th disease state is

$$\left[-\log_{2}p\left(d_{i}\right)\right]-[-\log_{2}p(d_{i}|r_{j})]=\log_{2}\frac{p(d_{i}|r_{j})}{p\left(d_{i}\right)},$$

with the expected value calculated in terms of the post-test distribution giving

(3) $$D(post||pre)=\overset{n}{\underset{i=1}{\sum }}p\left(d_{i}|r_{j}\right)\log_{2}\frac{p\left(d_{i}|r_{j}\right)}{p\left(d_{i}\right)}.$$

D(post||pre) is called the relative entropy (or the Kullback-Leibler divergence) from pre (the pretest probability distribution) to post (the posttest probability distribution) [23,24]. Its value is always nonnegative [23]. Relative entropy is sometimes thought of as a measure of distance from one probability distribution (pre) to another probability distribution (post). Since it is an asymmetric function, i.e., D(post||pre) and D(pre||post) are not necessarily equal, and because it does not satisfy the triangle inequality, it does not qualify as a true distance metric [23] (p.18). As illustrated by the above example, the expected value of the reduction in the surprisal (5/8 bits) is different than the reduction in the expected values of the surprisal (0 bits), i.e., the diagnostic information, in this case, is not simply pretest entropy minus posttest entropy. 

### 3.3. Mutual Information Quantifies the Diagnostic Information That We Expect to Receive by Testing

Using the same data set (Table 1) we consider the question of how much information we expect to receive if we randomly select and test an individual from this population. Hence, the question we are now asking is from the pretest perspective, in contrast to the posttest perspective of the preceding subsection. Once again, we quantify diagnostic information as the expected value of the reduction in the surprisal that results from testing. We found above that if the test result is $r_{3}$, then we obtain 5/8 = 0.625 bits of information. Using the relative entropy function (Equation (3)), we can also calculate that $r_{1}$ provides 0.227 bits of information and $r_{2}$ provides 0.343 bits of information. The probabilities of obtaining each of the three possible test results are 0.396, 0.438, and 0.167, respectively. Therefore, the amount of diagnostic information, on average, that we will receive by performing this test is

(0.396)(0.227 bits)+(0.438)(0.343 bits)+(0.167)(0.625 bits)=0.345 bits.

The expected value of the amount of diagnostic information to be obtained by testing is the expected value of the relative entropy. For the general case, this is

(4) $$I\left(D;R\right)=\overset{m}{\underset{j=1\;}{\sum }}p\left(r_{j}\right)\overset{n}{\underset{i=1}{\sum }}p(d_{i}|r_{j})\log_{2}\frac{p(d_{i}|r_{j})}{p\left(d_{i}\right)}=\overset{n}{\underset{i=1}{\sum }}\overset{m}{\underset{j=1}{\sum }}p\left(d_{i},r_{j}\right)\log_{2}\frac{p\left(d_{i},r_{j}\right)}{p\left(d_{i}\right)p\left(r_{j}\right)},\;\;\;$$

where $p\left(d_{i}\right)$ is the probability that a patient randomly selected from the population is in disease state $d_{i}$, $p\left(r_{j}\right)$ is the probability that a patient randomly selected from the population has test result $r_{j}$, and $p\left(d_{i},r_{j}\right)$ is the probability that a patient randomly selected from the population is both in disease state $d_{i}$ and has test result $r_{j}$. I(D;R) is known as the mutual information between the disease state and the test result [23]. It is called the mutual information between D and R because knowing value of D provides the same information about the value of R, on average, as knowing the value of R provides about the value of D, on average, i.e., I(D;R)=I(R;D).

Established consequences of the definitions of entropy and mutual information are that, for random variables X and Y,

(5) I(X;Y)=H(X)+H(Y)−H(X,Y),

and

(6) H(X|Y)=H(X,Y)−H(Y),

where H(X,Y) is the entropy of the random variable defined by the joint occurrence of the events defining X and Y and H(X|Y) is the entropy of the random variable defined by the events defining X conditional upon the events defining Y [23].

A consequence of Equations (5) and (6) is:

(7) H(D|R)=H(D)−I(D;R), 

i.e., performing a diagnostic test decreases the uncertainty about the disease state, on average, by the mutual information between D and R. Recall that, for the current example, H(D) = 1.75 bits and I(D;R) = 0.345 bits. Hence, the remaining uncertainty after performing this test is, on average, 1.405 bits. A perfect test would provide 1.75 bits of information.

In the preceding subsection we noted that relative entropy is not generally equal to pretest entropy minus posttest entropy. Here, however, where we are calculating the expected value of the amount of information that a test will provide, it is equal to pretest entropy minus posttest entropy: rearranging Equation (7) gives I(D;R) = H(D)−H(D|R).

The mutual information provided by a diagnostic test is a single parameter measure of the performance of the test. It is dependent upon the pretest probabilities of disease. What is known as the channel capacity is the maximum possible value of the mutual information across all possible distributions of pretest probabilities [23].

## 4. Quantifying the Diagnostic Information Provided by Two or More Tests

More than one diagnostic test is often required to characterize a patient’s disease state. In this section we extend the theory to situations in which more than one diagnostic test is performed.

### 4.1. Relative Entropy Applied to the Case of Multiple Diagnostic Tests

Let $p_{0}\left(d_{i}\right)$ be the pretest probability of the i-th disease state. Let $p_{a}\left(d_{i}\right)$ be the probability of the i-th disease state after performing test A and obtaining result $r_{a}$. Let $p_{b}\left(d_{i}\right)$ be the probability of the i-th disease state after performing test B and obtaining result $r_{b}$. Finally, let $p_{ab}\left(d_{i}\right)$ be the probability of the *i*-th disease state after performing both tests A and B and obtaining results $r_{a}$ and $r_{b}$. The amount of information provided by test A for the subgroup of patients with result $r_{a}$, as we saw in Section 3.2, is $D(p_{a}||p_{0})$. Similarly, the amount of information provided by test B for the subgroup of patients with result $r_{b}$ is $D(p_{b}||p_{0})$, and the amount of information provided by both tests for the subgroup of patients with results $r_{a}$ and $r_{b}$ is $D(p_{ab}||p_{0})$.

Now consider a patient belonging to the subset of patients with both result $r_{a}$ and result $r_{b}$. How much diagnostic information is obtained if only test A is performed? The reduction in the surprisal for the i-th disease state is

$$\left[-\log_{2}p_{0}\left(d_{i}\right)\right]-\left[-\log_{2}p_{a}\left(d_{i}\right)\right]=\log_{2}\frac{p_{a}\left(d_{i}\right)}{p_{0}\left(d_{i}\right)}.$$

To quantify the diagnostic information, we calculate the expected value of the reduction in the surprisal. Since the patient belongs to the subset of patients with results $r_{a}$ and $r_{b}$ the expectation is calculated using the $p_{ab}\left(d\right)$ distribution. This gives

(8) $$\underset{i}{\sum }p_{ab}\left(d_{i}\right)\log_{2}\frac{p_{a}\left(d_{i}\right)}{p_{0}\left(d_{i}\right)}.$$

We will call this the modified relative entropy (I. J. Good called this trientropy [25]). We can think of it as the distance from the $p_{0}\left(d\right)$ probability distribution to the $p_{a}\left(d\right)$ probability distribution when the true probability distribution is $p_{ab}\left(d\right)$. Expression (8) can yield negative diagnostic information values. This occurs when the pretest probability distribution is a better estimate of the true probability distribution than the posttest probability distribution. In Appendix A, we show that the modified relative entropy satisfies the triangle inequality but still fails to meet the criteria for a distance metric.

As an example of the application of Expression (8) to a case in which two diagnostic tests are performed, consider a situation in which a person is being evaluated for possible cancer. Assume two disease states, cancer and not cancer, and that a screening test increases the probability of this individual having cancer from 0.05 to 0.3, but that a subsequent, more definitive test, decreases the probability of cancer to 0.01. We can imagine that this person belongs to a theoretical population (A) in which 5% of its members have cancer. Screening identifies this patient as belonging to a subset (B) of A in which 30% of its members have cancer. Finally, the second test identifies the patient as belonging to a subset (C) of B in which 1% of its members have cancer. Using Expression (8) we calculate that the screening test provided −0.410 bits of information (*from* a probability of cancer of 0.05 *to* a probability of cancer of 0.3 given that the probability of cancer is actually 0.01) and that the second test provided 0.446 bits of information (*from* a probability of cancer of 0.3 *to* a probability of cancer of 0.01 given that the probability of cancer is actually 0.01). The two tests together provided 0.036 bits of information. We obtain this final value either by summing the information provided by each of the two tests or by calculating the relative entropy (Equation (3)) given the pretest probability of cancer of 0.05 and the posttest probability of cancer of 0.01. Although the screening test shifted the probability of cancer in the wrong direction for this specific individual, there is no reason to conclude that the result of the screening test was a mistake. The screening test did its job by properly identifying the individual as a member of subset B.

### 4.2. Mutual Information Applied to the Case of Multiple Diagnostic Tests

The mutual information common to random variables X, Y and Z is defined as

(9) I(X;Y;Z)=I(X;Y)−I((X;Y)|Z)

where I((X;Y)|Z)=I((X|Z);(Y|Z)) is the mutual information between and Y conditional upon Z [23] (p. 45). Hence, from Equations (5), (6), and (9):

(10) I(X;Y;Z)=H(X)+H(Y)+H(Z)−H(X,Y)−H(X,Z)−H(Y,Z)+H(X,Y,Z).

Although the mutual information between two random variables is always nonnegative, the mutual information among three random variables can be positive, negative, or zero [23] (p. 45).

The expected value of the amount of information that two diagnostic tests, A and B, will provide about the disease state is $I\left(D;\left(R_{A},R_{B}\right)\right)$. This can be expressed in terms of entropies (per Equation (5)) as

(11) $$I\left(D;\left(R_{A},R_{B}\right)\right)=H\left(D\right)+H\left(R_{A},R_{B}\right)-H\left(D,R_{A},R_{B}\right),$$

and it can be partitioned:

(12) $$I\left(D;\left(R_{A},R_{B}\right)\right)=I\left(D;R_{A}\right)+I\left(D;R_{B}\right)-I\left(D;R_{A};R_{B}\right).$$

Equation (12) can be proved by using Equations (5), (10), and (11) to replace the four mutual information terms with their entropy equivalents. Hence, the expected value of the information that tests A and B provide about disease state D is equal to the sum of the expected values of the information provided by each test minus $I\left(D;R_{A};R_{B}\right)$, a term that quantifies the interaction among D, $R_{A}$, and $R_{B}$. Since $I\left(D;R_{A};R_{B}\right)$ can be positive, negative, or zero, $I\left(D;\left(R_{A},R_{B}\right)\right)$ can be less than, greater than, or equal to the sum of $I\left(D;\left(R_{A}\right)\right)$ and $I\left(D;\left(R_{B}\right)\right)$, respectively.

Alternatively, we can use Equations (9) and (12) to partition $I\left(D;\left(R_{A},R_{B}\right)\right)$ as follows:

(13) $$I\left(D;\left(R_{A},R_{B}\right)\right)=I\left(D;R_{A}\right)+I\left(\left(D;R_{B}\right)|R_{A}\right),$$

where $I(\left(D;R_{B}\right)|R_{A})=I\left(D|R_{A};R_{B}|R_{A}\right)$ is the average incremental information provided by test B after performing test A. $I(\left(D;R_{B}\right)|R_{A})$ can be expressed in terms of entropies using Equations (5) and (6):

(14) $$I(\left(D;R_{B}\right)|R_{A})=H\left(D,R_{A}\right)+H\left(R_{A},R_{B}\right)-H\left(R_{A}\right)-H\left(D,R_{A},R_{B}\right).$$

Although the expressions become more complicated as the number of diagnostic tests increase, the mutual information between the disease state and the results of multiple diagnostic tests can be partitioned in fashions analogous to Equations (12) and (13). For the case in which there are three diagnostic tests:

(15) $$\begin{array}{c} I\left(D;\left(R_{A},R_{B},R_{C}\right)\right)=I\left(D;R_{A}\right)+I\left(D;R_{B}\right)+I\left(D;R_{C}\right)-I\left(D;R_{A};R_{B}\right)-I\left(D;R_{A};R_{C}\right)\\ -I\left(D;R_{B};R_{C}\right)+I\left(D;R_{A};R_{B};R_{C}\right)\\ =I\left(D;R_{A}\right)+I(\left(D;R_{B}\right)|R_{A})+I(\left(D;R_{C}\right)|\left(R_{A},R_{B}\right)). \end{array}$$

These equations can be proven, once again, by replacing the mutual information terms with their entropy equivalents, recognizing that

(16) $$I\left(D;R_{A};R_{B};R_{C}\right)=I\left(D;R_{A};R_{B}\right)-I(\left(D;R_{A};R_{B}\right)|R_{C})$$

The entropy function, expressed as Equation (2), is not defined for continuous random variables. Nevertheless, the mutual information between or among continuous random variables, which is defined, can be approximated numerically as the sum and differences of entropies using Equations (5), (10), (11), and (14) [23] (pp. 231–232). 

## 5. Historical Background

With this understanding of basic information theory functions, we can briefly consider the development of information theory and the evolution of its application to a clinical diagnostic testing. The concept of entropy is probably most familiar within the context of thermodynamics, where it is a measure of the “degree of randomness” of a physical system [18] (p. 12). Although an understanding of the basic principles of thermodynamics preceded the development of information theory, the entropy of thermodynamics can be understood to be an application of the concept of entropy stated by Equation (2). The difference between the two functions is that in thermodynamics Equation (2) is multiplied by the Boltzmann constant to provide the appropriate physical dimensions (joules per kelvin) [26] (p. 30).

As mentioned in Section 1, Hartley and Shannon were early developers of information theory. Hartley published a paper in 1928 concerning the relationship between the quantity of information transmitted over a system and the width of the frequency range of the transmission [1]. He defined entropy (which he called information) for situations in which the possible states are equally likely. The more general concept of entropy, stated by Equation (2), was defined in 1948 by Shannon in “A Mathematical Theory of Communication” [2]. This foundational paper also defined mutual information and channel capacity. Relative entropy was introduced by Kullback and Leibler in 1951 [24]. 

The applicability of information theory to a clinical diagnostic testing was not immediately recognized, has been slow in its development, and remains an area of research. As noted in the introduction, Good and Card probably published the first paper on the subject in 1971 [3]. Their contribution was not recognized by many subsequent authors interested in this subject. To a large extent, the history of the application of information theory to clinical diagnostic testing is the history of the discovery of concepts previously understood by Good and Card. They recognized that mutual information (what they called mean information transfer) quantifies the expected value of the amount of information provided by a test and that this function can be used regardless of the number of disease states and test results. Implicit in their report is the use of relative entropy and, what we have called, modified relative entropy (in their language, dinegentropy and trientropy, respectively) to quantify the information provided by specific test results. They also quantified the information gained by sequential testing [3,27].

The “weight of evidence in favor of a hypothesis” is a central concept in the Good and Card paper [3]. The concept was developed independently by C.S. Peirce [28] and A.M. Turing (possibly in collaboration with I.J. Good) [29]. The weight of evidence in favor of disease state $d_{i}$ given result $r_{j}$, as opposed to the other disease states, ${\bar{d}}_{i}$ can be expressed as

log[p(rj|di)p(rj|d¯i)].

This is equal to

$$\begin{array}{c} \log\frac{p\left(d_{i}|r_{j}\right)}{p\left(d_{i}\right)}-\log\frac{p\left({\bar{d}}_{i}|r_{j}\right)}{p\left({\bar{d}}_{i}\right)}\\ =[-\log p\left(d_{i}\right)-\;-\log p(d_{i}|r_{j})]-[-\log p\left({\bar{d}}_{i}\right)-\;-\log p({\bar{d}}_{i}|r_{j})]. \end{array}$$

As pointed out by Good and Card, we find by looking at each of the above two expressions in brackets (which are reductions in surprisals) that weight of evidence can be interpreted in terms of quantities of information; in this case, as the amount of information that $r_{j}$ provides about $d_{i}$ minus the amount of information that $r_{j}$ provides about ${\bar{d}}_{i}.$ A second important observation about the weight of evidence is that it is equal to the logarithm of a likelihood ratio. This point has been used to advantage by Van den Ende et al. to provide clinicians with an accessible approach to interpreting diagnostic tests, including the fact that the logarithm of the pretest odds plus the weight of evidence equals the logarithm of the posttest odds [30]. Since weight of evidence can be interpreted in terms of quantities of information, the logarithm of a likelihood ratio is an information quantity and so has information units. When working in log base 10 (as in [30]) the appropriate unit is the hartley. To convert from hartleys to bits, divide by $\log_{10}2=0.301.$

Most papers on the application of information theory to clinical diagnostic testing are founded upon a report published by Metz, Goodenough, and Rossmann in 1973 [4]. They derived the expression for the information content (mutual information) of a diagnostic test as a function of the pretest probability of disease and the test’s true positive rate (probability of a positive result given disease) and false positive rate (probability of a positive result given no disease), i.e., they used these parameters to calculate the posttest probability distribution and then used the pretest and posttest distributions to calculate mutual information. They applied the theory to the evaluation of radiographic systems and noted that this statistic can be used to compare points on the same or different receiver operating characteristic (ROC) curves (defined below in Section 6.1) [31]. The area under the ROC curve (AUC) is a popular measure of diagnostic test performance [32]. Relationships between the AUC and mutual information are discussed in the example presented in Section 6.1. Metz et al. also suggested that the performance of a diagnostic test be quantified as the maximum of the set of information contents associated with the points on a test’s ROC curve ($I_{max})$. Subsequent authors suggested that $I_{max}$ can be used in the selection of the point that partitions test results into normal results and abnormal results [5,7,33], i.e., the diagnostic cutoff. The use of a diagnostic cutoff, however, can result in some loss of diagnostic information [6,34,35]. This is illustrated by examples presented in Section 6.2 and Section 6.3.

Diamond and colleagues applied information theory in 1981 to the quantification of the performance of the exercise electrocardiogram (ECG) in the diagnosis of coronary heart disease (CHD) [6]. This paper is discussed in Section 6.2. The primary theoretical contribution of their paper is the recognition that it is not necessary to select a single diagnostic cutoff in order to calculate the information content (mutual information) provided by a diagnostic test. This concept is implicit in the work of Good and Card [3].

The relative entropy function was applied to clinical diagnostic testing in 1999 by Lee [36] and, independently, by Benish [8]. Lee used the relative entropy between the distributions of test results for diseased subjects and disease-free subjects to characterize the potential of a diagnostic test to rule in (confirm) and rule out (exclude) disease. A different approach to characterizing the potential of a diagnostic test to rule in or rule out disease states is illustrated by examples presented in Section 6.2 and Section 6.4. Benish recognized that the relative entropy function allows for calculation of the information provided by a specific test result. Once again, this observation is implicit in the paper by Good and Card [3]. Use of the relative entropy function for this purpose is discussed above in Section 3.2 and Section 4.1 and is demonstrated in Section 6.2, Section 6.4, and Section 6.5. Benish also discussed the channel capacity of a medical diagnostic test [37]. Hughes, writing from the perspective of a plant disease epidemiologist, published the only book on the application of information theory to diagnostic testing in 2012 [9].

Section 4, above, develops concepts found in the work by Good and Card regarding the quantification of information provided by multiple diagnostic tests [3,27]. These functions are illustrated in the examples presented in Section 6.4 and Section 6.5.

## 6. Examples

R code for the calculations and figures in the following examples are available in the Supplementary Materials. 

### 6.1. The Relationship between I(D;R) and the AUC

ROC curves are often used to describe the performance of a diagnostic test when the test results lie on a continuum or are otherwise ordered [31]. This methodology is applicable when two disease states are under consideration, e.g., disease present and disease absent. A ROC curve plots the tradeoff between the true positive rate (test sensitivity) and the false positive rate (1 − test specificity) as the cutoff point for defining normal and abnormal test results is moved along the ordered set of results. As noted above, the AUC is a popular measure of diagnostic test performance [32]. Both the AUC and I(D;R) are single-parameter measures of diagnostic test performance. It is helpful to understand some of their differences.

**Figure 2:**
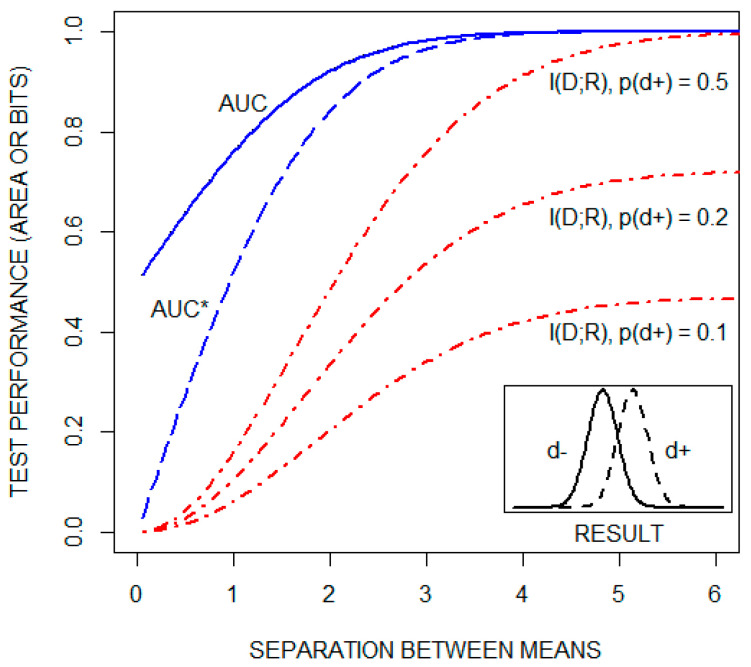
The area under the receiver operating characteristic curve (AUC), a transformation of the AUC (AUC*), and the mutual information between the disease state and the test result (I(D;R) in units of bits), as a function of the distance between the means of the distributions of test results for both healthy (d−) and diseased (d+) individuals (see text). I(D;R) is plotted for three pretest probabilities of disease: 0.1, 0.2, and 0.5.

A classic approach to explaining ROC curves is to assume that test results are normally distributed for both healthy (d−) and diseased (d+) individuals. This is illustrated by the Figure 2 insert. The ROC curve is then constructed, as noted above, by plotting test sensitivity as a function of 1-test specificity for all possible diagnostic cutoffs. As the distance between the means of the two distributions increases, the ROC curve shifts upward and to the left, increasing the AUC from a value of 0.5 toward its maximal value of one. This is illustrated in Figure 2, which includes a plot of the AUC as a function of the separation between the means, for the case in which the standard deviations of both distributions are one. I(D;R), but not the AUC, is a function of the pretest probability of disease. This is illustrated in the figure by plots of I(D;R) as a function of the distance between the means of the same two distributions for three pretest probabilities of disease: 0.1, 0.2, and 0.5. The figure also plots a transformation of the AUC, AUC*, which is equal to 2(AUC)−1. This transformation of the AUC changes its range from [0.5,1] to [0,1]. Collectively, these plots demonstrate that the AUC and I(D;R) are qualitatively different statistics.

### 6.2. Diagnostic Information from the Exercise Electrocardiogram (ECG) 

As noted in the preceding section, Diamond et al. used information theory to evaluate the performance of the exercise ECG in the diagnosis of CHD [6]. Depression of the ST segment (a portion of the ECG tracing) during exercise is an indicator of coronary artery disease. The data in Table 2 shows their estimates of the probability of ST segment depression falling into six different categories as a function of whether the patient has significant CHD.

They first analyzed the data by selecting a criterion to dichotomize the results into positive and negative categories. For example, if a positive test is defined as ST depression ≥ 1 mm, then, as seen from the table, p(r+|d+) becomes 0.233 + 0.088 + 0.133 + 0.195 = 0.649 and p(r+|d−) becomes 0.110 + 0.021 + 0.012+ 0.005 = 0.148. Recognizing that $p\left(d_{i},r_{j}\right)=p\left(r_{j}|d_{i}\right)p\left(d_{i}\right)$ and $p\left(r_{j}\right)=p\left(d+,r_{j}\right)+p\left(d-,r_{j}\right),$ Equation (4) can then be used to calculate the information content (mutual information) for the test for this cutoff as a function of the pretest probability of disease.

They contrast this with a calculation of the information content (mutual information) if the results are not dichotomized, but rather left partitioned into six categories. If the ST segment is depressed by 2.2 mm for example, it makes sense to calculate the posttest probability using the more accurate test operating characteristics that apply to the narrower interval of [2, 2.5) than the operating characteristics that apply to the larger interval of [1, ∞). Equation (4) is again used to make the calculation, but in this case, there are six possible test results rather than two.

**Figure 3:**
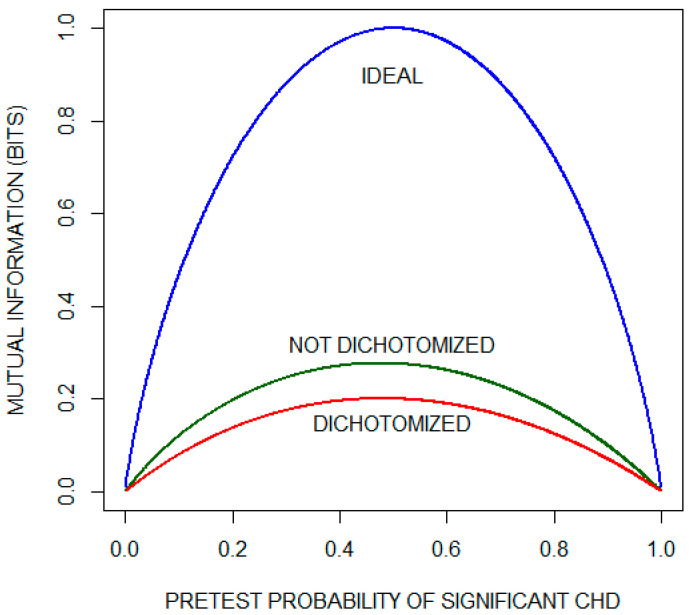
Mutual information as a function of pretest probability of significant coronary heart disease (CHD) for the exercise electrocardiogram. The plot compares the performance of a theoretical ideal test with the actual performance when the results are either (1) dichotomized using the criterion of ST segment depression of ≥ 1 mm or (2) not dichotomized. This plot has been reconstructed with permission from the paper by Diamond et al. [6].

Figure 3 (reconstructed from their report with permission) compares mutual information as a function of pretest probability of significant CHD for the dichotomized and non-dichotomized approaches. The curve labeled IDEAL is the pretest diagnostic uncertainty as a function of pretest probability. It indicates the average amount of information that an ideal test would provide, i.e., the average amount of information needed to reduce the diagnostic uncertainty to zero (by yielding a posttest probability of either zero or one). We observe that, for most of the range of pre-test probabilities, approximately one third of the diagnostic information is lost by dichotomizing the results with a diagnostic cutoff of 1 mm. The issue of information lost as a consequence of dichotomizing test results is considered again in the following subsection. 

**Figure 4:**
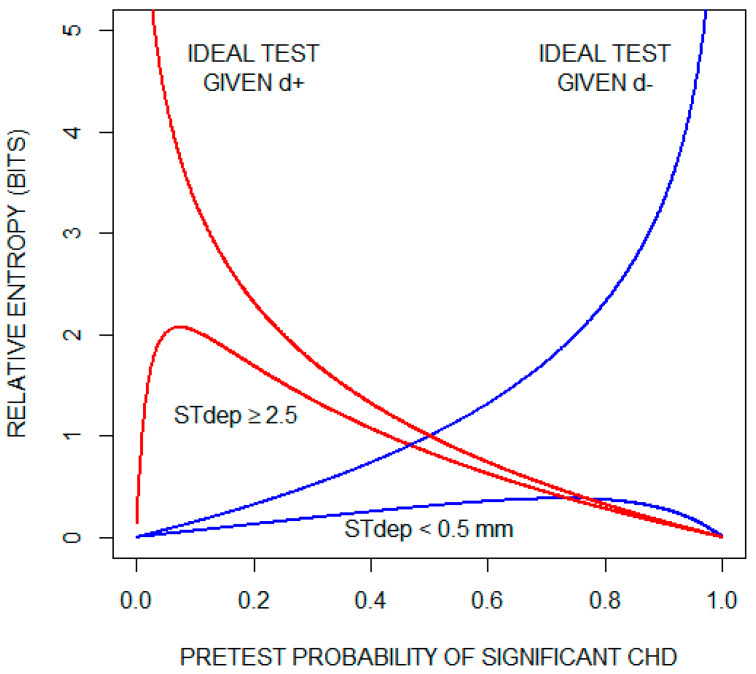
Diagnostic information (relative entropy) provided by the findings of an ST segment depression (STdep) < 0.5 mm and an ST depression (STdep) ≥ 2.5 mm as a function of pretest probability of significant coronary heart disease (CHD). Also shown are relative entropy plots for a theoretical ideal test when significant CHD is present (d+) and when significant CHD is absent (d−). For the theoretical ideal test when significant CHD is present, relative entropy increases indefinitely as pretest probability of significant CHD approaches zero; and for the theoretical ideal test when significant CHD is absent, relative entropy increases indefinitely as pretest probability of significant CHD approaches one.

Although, on average, the exercise ECG does not provide much information about whether a patient has significant CHD, the possibility remains that specific test results are informative. To illustrate this, we consider the two results that lie on opposite ends of the test result spectrum: ST depression < 0.5 mm and ST depression ≥ 2.5 mm. Recall that relative entropy (Equation (3)) quantifies the amount of diagnostic information provided by a given test result. Figure 4 plots relative entropy as a function of the pretest probability of significant CHD for these two test results. For comparison, the figure includes relative entropy plots for a theoretical ideal test when significant CHD is present (d+) and when significant CHD is absent (d−). Inspecting these curves, we conclude that an ST depression of < 0.5 mm is not helpful in ruling out significant CHD. On the other hand, when significant CHD is present and as the pre-test probability increases, the information provided by an ST depression of ≥ 2.5 mm approaches the information provided by the ideal test.

### 6.3. Diagnostic Information Lost by Selecting a Diagnostic Cutoff 

Diagnostic test results are often continuous or lie along a continuum, e.g., body temperature, serum glucose, histologic grade. As observed in the preceding example, dichotomizing test results by selecting a diagnostic cutoff can result in some loss of diagnostic information. As an additional illustration of this, consider again the classic example in which the probability densities of test results are normally distributed for both the healthy (d−) and diseased (d+) populations (as illustrated by the Figure 2 insert). Let us assume that the pretest probability of disease is 0.2, that the standard deviations of the two distributions are equal to one, and that the means of the two distributions are separated by one standard deviation.

The cutoff that maximizes the mutual information provided by this test lies between the means of the two distributions, approximately 0.66 standard deviations from the mean of the healthy population (determined by inspection; see the Supplementary Materials). This results in a test sensitivity, p(r+|d+), of 0.63 and a test specificity, p(r−|d−), of 0.75. Recalling that $p\left(d_{i},r_{j}\right)=p(r_{j}|d_{i})p\left(d_{i}\right)$ and $p\left(r_{j}\right)=p\left(d+,r_{j}\right)+p\left(d-,r_{j}\right)$, Equation (4) can be used to calculate that the average amount of information gained by performing the test with this cutoff is 0.071 bits.

Alternatively, we can calculate the posttest probabilities directly from the obtained results. Since the test results are continuous, we modify Equation (4) to calculate I(D;R) as follows:

$$\overset{2}{\underset{i=1}{\sum }}\int_{-\infty }^{\infty }\rho \left(d_{i},r\right)\log_{2}\frac{\rho \left(d_{i},r\right)}{p\left(d_{i}\right)\rho \left(r\right)}dr.$$

Because *r* is a continuous variable, we have replaced the summation over index *j* with an integral and, for terms involving *r*, replaced the probabilities (indicated by *p*) with probability densities (indicated by *ρ*). Note that *ρ*$\left(d_{i},r\right)$ = *ρ*$(r|d_{i})p\left(d_{i}\right)$ and *ρ*(r) = *ρ*(d+,r) + *ρ*(d-,r). This calculation gives a mutual information of 0.106 bits. Hence, in this example, approximately one third of the expected value of the information provided by the test is discarded by selecting a cutoff to dichotomize the results.

### 6.4. Diagnostic Information Provided by Two Tests with Discrete Results

A study that investigated the value of combining two diagnostic tests in the diagnosis of deep vein thrombosis (DVT) [38] provides a convenient data set to illustrate information theory functions that apply when more than one test is used (see Section 4). A DVT is a blood clot of the deep veins, typically in the lower extremities, that can be fatal if it detaches and travels to the lungs. One of the tests is a clinical index, based on the patient’s medical history and physical exam findings, that classified the patient as being at low, moderate, or high risk for a DVT. The other test is a blood test that detects a protein, the d-dimer, that is often elevated in the presence of a DVT. The d-dimer was reported as positive or negative. The number of patients found to be in each of the 3 × 2 test result categories as a function of whether they were ultimately diagnosed with a DVT is shown in Table 3.

The study included 1057 patients, 190 of whom were diagnosed as having a DVT. Therefore, the probability of being diagnosed with a DVT in this population is 190/1057 = 0.180. The uncertainty about whether a patient randomly selected from this population was diagnosed with a DVT is calculated using the entropy function (Equation (2)). We find that H(D)=0.680 bits. Given that only two disease states are under consideration, the range of possible entropy values is 0–1 bits.

If the clinical index (test A) is applied as a single test within this population, the diagnostic uncertainty will decrease, on average, by $I\left(D;R_{A}\right)=0.111$ bits (calculated using Equation (5)). Similarly, the d-dimer test (test B) will decrease the diagnostic uncertainty, on average, by $I\left(D;R_{B}\right)=0.125$ bits. Using the information provided by both tests will decrease the diagnostic uncertainty, on average, by $I\left(D;(R_{A},R_{B})\right)=0.197$ bits (calculated using Equation (11)), which is less than the sum of the information provided by each test separately by a value of $I\left(D;R_{A};R_{B}\right)=0.039$ bits (see Equation (12); this value was calculated using Equation (10)). The residual uncertainty after performing both tests is substantial: $H\left(D\right)-I\left(D;(R_{A},R_{B})\right)=0.483$ bits (an uncertainty reduction of 29%). A perfect test or combination of tests would reduce the uncertainty to zero.

If the clinical index is found to be high and no additional testing is performed, the posttest probability of a DVT is 0.472. Using the relative entropy function (Equation (3)), we calculate that the test has provided 0.323 bits of information. An isolated negative d-dimer yields a posttest probability of a DVT of 0.052 and 0.106 bits of information. The range of possible relative entropy values is bounded by the relative entropies associated with reducing the diagnostic uncertainty to zero. For this example, 2.476 bits of information are required to rule in a DVT (going from the pretest probability of 0.180 to a posttest probability of one) and 0.286 bits of information are required to rule out a DVT (going from the pretest probability of 0.180 to a post-test probability of zero). Using the pre-test probabilities and these boundary information values, we calculate that the expected value of the amount of information provided by a theoretical perfect test is (0.180)(2.476 bits)+(0.820)(0.286 bits)=0.680 bits=H(D).

Next, consider a patient who belongs to the subset of patients with both a high clinical index and a negative d-dimer. The probability of a DVT given both results is 0.231 and, per the relative entropy function (Equation (3)), this combination of results provides 0.012 bits of information. Because the two test results are discordant, their net effect is to provide very little diagnostic information. Furthermore, by applying Expression (8) we find that for this subpopulation (patients with a high clinical index and a negative d-dimer) the information provided by each of the two tests performed separately is negative (−0.168 bits for the finding of a high clinical index and −0.254 bits for the finding of a negative d-dimer). The negative results indicate that the baseline probability of a DVT is a more accurate estimate than the posttest probability when only one test is performed.

Although these two tests do not, on average, provide much information about whether a patient has a DVT, they have the potential to help rule out a DVT. We saw above that an isolated negative d-dimer decreases the probability of a DVT from 0.180 to 0.052 and provides 0.106 bits of information. An isolated low clinical index decreases the probability to 0.045 and provides 0.120 bits of information. The combination of both findings decreases the probability to 0.009 and provides 0.229 bits of information (out of the 0.286 bits of information required to decrease the probability of a DVT to zero).

### 6.5. Diagnostic Information Provided by Two Tests with Continuous Results 

Finally, we consider a hypothetical example in which the results of two diagnostic tests (A and B) are normally distributed for both healthy and diseased patients. We define the means, variances, and covariance for the binormal distribution of the test results for the healthy population as:

$$\mu_{A}=0,\;\mu_{B}=0,\;\sigma_{A}^{2}=1,\;\sigma_{B}^{2}=2,\;\sigma_{AB}^{2}=1,$$

and the parameters for the binormal distribution of the test results for the diseased population as:

$$\mu_{A}=2,\;\mu_{B}=1,\;\sigma_{A}^{2}=2,\;\sigma_{B}^{2}=1,\;\sigma_{AB}^{2}=-1.$$

**Figure 5:**
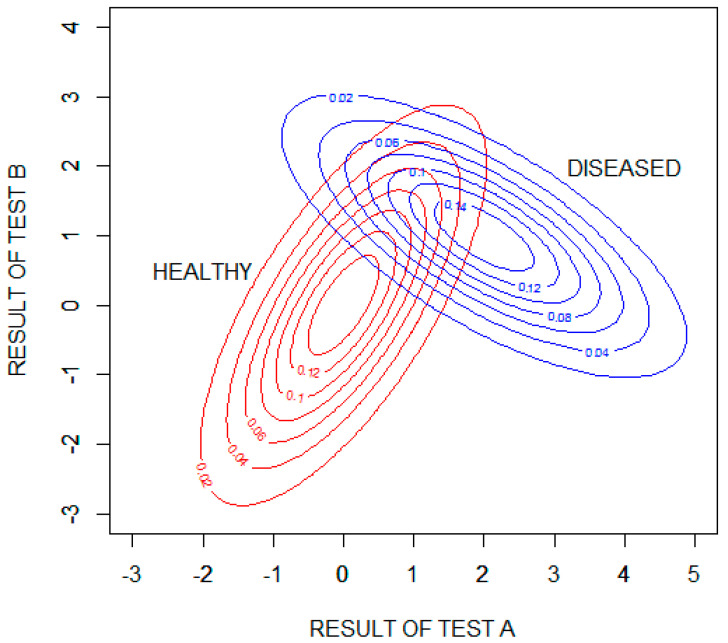
Contour plots showing the probability densities of the results of tests A and B for the healthy and diseased populations. The test results are expressed as standard deviations from the means of the distribution for healthy patients $\left(\mu_{A}=\mu_{B}=0\right)$.

Figure 5 shows probability density contour plots for the two binormal distributions, with test results expressed as standard deviations from the means of the distribution for healthy patients $\left(\mu_{A}=\mu_{B}=0\right)$. 

We will assume that the pretest probability of disease is 0.2. The pretest uncertainty of the disease state, H(D), is then found to be 0.722 bits (calculated using Equation (2)). Test A applied as a single test within this hypothetical population will decrease the diagnostic uncertainty, on average, by $I\left(D;R_{A}\right)$ = 0.281 bits (calculated numerically using Equation (5)). Test B applied as a single test within this population will decrease the diagnostic uncertainty, on average, by $I\left(D;R_{B}\right)$ = 0.083 bits. Test A is, on average, more informative than test B because the separation between the means of the healthy and diseased populations is larger for test A than for test B. Using the information provided by both tests will decrease the diagnostic uncertainty, on average, by $I\left(D;\left(R_{A},R_{B}\right)\right)$ = 0.424 bits (calculated numerically using Equation (11)), which is more than the sum of the information provided by each test separately by a value of $-I\left(D;\left(R_{A},R_{B}\right)\right)$ = 0.060 bits (see Equation (12); this value was calculated numerically using Equation (10)). The residual uncertainty after performing both tests is: $H\left(D\right)\;-\;I\left(D;\left(R_{A},R_{B}\right)\right)\;=\;0.298$ bits (an uncertainty reduction of 59%).

**Figure 6:**
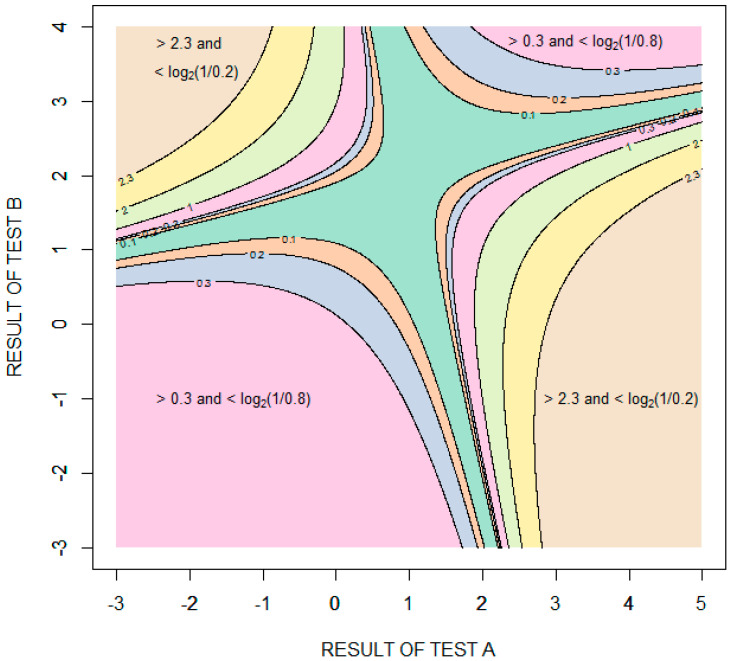
Contour plot showing the information (relative entropy in units of bits) provided by specific combinations of results of tests A and B. The test results are expressed as standard deviations from the means of the distribution for healthy patients $\left(\mu_{A}=\mu_{B}=0\right)$.

Figure 6 is a contour plot showing the amount of diagnostic information (relative entropy in units of bits) provided by possible combinations of the results of the two tests, with test results expressed as standard deviations from the means of the distribution for healthy patients $\left(\mu_{A}=\mu_{B}=0\right)$. The information value is bounded in two opposing quadrants by the amount of information required to rule in disease, $\log_{2}\left(1/0.2\right)\approx 2.322$ bits, and in the other two opposing quadrants by the amount of information required to rule out disease, $\log_{2}\left(1/0.8\right)\approx 0.322$ bits. More information is required to rule in disease than to rule out disease because the pretest probability of disease is less than 0.5. Given that the performance of the tests is characterized by binormal distributions, it is theoretically impossible to rule in or rule out disease with certainty. In the case of ruling in disease, for example, as the results of test A increase and the results of test B decrease, the probability of disease approaches (but never equals) one and the amount of information obtained approaches (but never equals) $\log_{2}\left(1/0.2\right)$ bits.

## 7. Discussion

### 7.1. Summary 

Information theory functions are defined on random variables. The recognition that an individual’s disease state and diagnostic test result are random variables allows for the direct application of fundamental information theory functions to clinical diagnostic testing. The concept of the surprisal (discussed in Section 2) provides an intuitive understanding of these functions. The uncertainty about a patient’s disease state is quantified by the entropy function (discussed in Section 3.1). The amount of diagnostic information provided by a specific test result is quantified by the relative entropy function (discussed in Section 3.2). Prior to performing a diagnostic test, the expected value of the amount of information that the test will provide is quantified by the mutual information function (discussed in Section 3.3). The mutual information associated with a diagnostic test is a single parameter measure of diagnostic test performance that is dependent upon the pretest probabilities of the disease states. The information theory functions are applicable given any number of disease states and any number of test results (or continuous test results). Information theory functions can also be used to evaluate the conjoint performance of multiple diagnostic tests: the information provided by each of several test results can be calculated (discussed in Section 4.1), and the mutual information between the disease state and the results of multiple diagnostic tests can be partitioned into components corresponding to the contributions of the individual tests and interactions among the disease states and test results (discussed in Section 4.2).

### 7.2. Points of Emphasis and Clarification

- Diagnostic information does not necessarily decrease diagnostic uncertainty. A screening test, for example, might change the probability of disease from 0.005 to 0.5. The test has provided 2.826 bits of information (quantified by the relative entropy function), while the uncertainty has increased from 0.045 bits to one bit (quantified by the entropy function). This is consistent with the common experience of becoming more perplexed about an issue as more is learned about it.
- The three questions posed in the Introduction all include the word “we”, implying a general agreement upon the probabilities used to calculate diagnostic uncertainty and diagnostic information. This is often not the case. Probability estimates usually include a subjective component. Consequently, two individuals can obtain different amounts of information from the same test result or observation.
- Mutual information, but not relative entropy, is equal to pretest uncertainty minus posttest uncertainty. Both functions are equal to the expected value of the reduction in the surprisal.
- The random variables of interest in this review are an individual’s disease state and test result. The same theory applies when the random variables are defined as any type of state and any type of observation.
- The goal of clinical diagnostic testing is not to make the diagnosis but, rather, to assign accurate probabilities to the possible disease states.
- Some diagnostic information is typically lost by dichotomizing continuous or ordered test results.

### 7.3. Conclusions

Information statistics have a useful role to play in the evaluation and comparison of diagnostic tests. In some cases, information measures may complement useful concepts such as test sensitivity, test specificity, and predictive values. In other situations, information measures may replace more limited statistics. Mutual information, for example, may be better suited as a single parameter index of diagnostic test performance than alternative statistics. Furthermore, information theory has the potential to help us learn about and teach about the diagnostic process. Examples include concepts illustrated above, including the importance of pretest probability as a determinant of diagnostic information, the amount of information lost by dichotomizing test results, the limited potential of some diagnostic tests to reduce diagnostic uncertainty, and the ways in which diagnostic tests can interact to provide diagnostic information. These are concepts that can all be effectively communicated graphically. 

It is hoped that this review will help to motivate new applications of information theory to clinical diagnostic testing, especially as data from newer diagnostic technologies becomes available. The challenge will be to develop systems that accurately diagnosis and treat patients by integrating increasingly large amounts of personalized data [39,40]. A potential role for information theory functions in this process is suggested by their applicability to multidimensional data.

## Figures and Tables

**Table 1:** Hypothetical data showing the number of individuals in a given disease state ($d_{1},\;d_{2},\;d_{3},$ or $d_{4}$) and with a given test result ($r_{1},r_{2},$ or $r_{3})$.

	$d_{1}$	$d_{2}$	$d_{3}$	$d_{4}$	
$r_{1}$	8	24	4	2	38
$r_{2}$	2	20	0	20	42
$r_{3}$	2	4	8	2	16
	12	48	12	24	96

**Table 2:** Data from Diamond et al. [6] showing the probabilities of various categories of ST segment depression (the result, *r*) during an exercise electrocardiogram as a function of the presence (d+) and absence (d−) of significant CHD.

ST Depression (mm)	p(r|d+)	p(r|d−)
0≤ST<0.5	0.143	0.625
0.5≤ST<1.0	0.208	0.227
1.0≤ST<1.5	0.233	0.110
1.5≤ST<2.0	0.088	0.021
2.0≤ST<2.5	0.133	0.012
2.5≤ST<∞	0.195	0.005

**Table 3:** Data from Anderson et al. [38]. The number of patients with and without a DVT as a function of the clinical index and the d-dimer test.

Clinical Index	d-Dimer	DVT+	DVT−
Low risk	−	3	313
	+	17	113
Moderate risk	−	15	243
	+	61	93
High risk	−	15	50
	+	79	55

## Supplementary Materials

R code for calculations and figures are available online at https://www.mdpi.com/1099-4300/22/1/97/s1.

## Appendix A. Modified Relative Entropy Is not a Distance Metric

We first show that the modified relative entropy function (Expression (8)) satisfies the triangle equality. Let the modified relative entropy from probability distribution b to probability distribution a when the true probability distribution is c be expressed as

$$d_{c}\left(a,b\right)=\underset{i}{\sum }c_{i}\log\frac{a_{i}}{b_{i}}.$$

Then,

$$d_{c}\left(x,y\right)+d_{c}\left(y,z\right)=\underset{i}{\sum }c_{i}\;\log\frac{x_{i}}{y_{i}}+\overset{}{\underset{i}{\sum }}c_{i}\;\log\frac{y_{i}}{z_{i}}=\overset{}{\underset{i}{\sum }}c_{i\;}\log\frac{x_{i}}{z_{i}}=d_{c}\left(x,z\right).$$

Despite satisfying the triangle inequality, $d_{c}\left(a,b\right)$ does not meet the criteria for a distance metric [41] (p. 117) because it can be negative. If we try to circumvent this problem by defining the measure as the absolute value of $d_{c}\left(a,b\right),$ then the triangle inequality is still satisfied:

$$\left|d_{c}\left(x,y\right)\right|+\left|d_{c}\left(y,z\right)\right|\geq \left|d_{c}\left(x,y\right)+d_{c}\left(y,z\right)\right|=|d_{c}\left(x,z\right)|.$$

Nevertheless, $\left|d_{c}\left(a,b\right)\right|$ still fails to qualify as a distance metric because it is not necessarily the case that $\left|d_{c}\left(a,b\right)\right|=0$ implies that a=b. For example, if $a_{1}=b_{2}=1/4\;$, $a_{2}=b_{1}=3/4$, and $c_{1}=c_{2}=1/2$, then $\left|d_{c}\left(a,b\right)\right|=0$ but a≠b.
